# Pediatric versus adult high grade glioma: Immunotherapeutic and genomic considerations

**DOI:** 10.3389/fimmu.2022.1038096

**Published:** 2022-11-22

**Authors:** Payal Aggarwal, Wen Luo, Katherine C. Pehlivan, Hai Hoang, Prajwal Rajappa, Timothy P. Cripe, Kevin A. Cassady, Dean A. Lee, Mitchell S. Cairo

**Affiliations:** ^1^ Department of Pediatrics, New York Medical College, Valhalla, NY, United States; ^2^ Department of Pathology, Microbiology and Immunology, New York Medical College, Valhalla, NY, United States; ^3^ Center for Childhood Cancer Research, Abigail Wexner Research Institute at Nationwide Children’s Hospital, Columbus, OH, United States; ^4^ Department of Medicine, New York Medical College, Valhalla, NY, United States; ^5^ Department of Cell Biology and Anatomy, New York Medical College, Valhalla, NY, United States

**Keywords:** high grade glioma, glioblastoma, pediatric, adult, immunotherapy

## Abstract

High grade gliomas are identified as malignant central nervous tumors that spread rapidly and have a universally poor prognosis. Historically high grade gliomas in the pediatric population have been treated similarly to adult high grade gliomas. For the first time, the most recent classification of central nervous system tumors by World Health Organization has divided adult from pediatric type diffuse high grade gliomas, underscoring the biologic differences between these tumors in different age groups. The objective of our review is to compare high grade gliomas in the adult versus pediatric patient populations, highlighting similarities and differences in epidemiology, etiology, pathogenesis and therapeutic approaches. High grade gliomas in adults versus children have varying clinical presentations, molecular biology background, and response to chemotherapy, as well as unique molecular targets. However, increasing evidence show that they both respond to recently developed immunotherapies. This review summarizes the distinctions and commonalities between the two in disease pathogenesis and response to therapeutic interventions with a focus on immunotherapy.

## 1 Introduction

High Grade Gliomas (HGG) represent the most common and aggressive brain tumor noted to have poor outcomes in adults and children (1–3). In the adult population, HGG represents the majority of primary brain tumors and therefore been extensively studied over many years (1–3). By contrast, pediatric high grade gliomas (pHGG) have remained a relatively under-investigated disease, as they represent only a fraction of primary malignant brain tumors in children. Recent genomic and epigenomic profiling studies have revealed key distinctions between adult HGG (aHGG) and pHGG, suggesting they are quite different diseases (1–3).

In fact, the 2021 WHO Classification of Tumors of the Central Nervous System (CNS), also referred to as WHO CNS 5, included major changes to the categorization of high grade gliomas and separated pHGG from aHGG to reflect this concept of their differing biology (4). Another major change in the WHO CNS 5 affecting the diagnostic implications for high grade gliomas is that tumors are now graded within tumor entities, while in past versions, a single diagnostic entity corresponded to specific tumor grade. This slightly complicates the delineation of low grade vs. high grade glioma, as previously it was clear from the tumor diagnostic entity whether it was low grade (generally considered Grade 1 or 2) vs. high grade (generally grade 3 or 4). Furthermore, in this version, both molecular and histologic findings may impact tumor grade within a given entity, with certain molecular alterations automatically designating a high grade tumor regardless of histologic findings (4).

When compared to the WHO CNS 4, published in 2016, WHO CNS 5 shows a continued progression towards an integrated molecular and histological diagnostic approach. Whereas in the WHO CNS 4, glioblastomas (GBMs) included both IDH- mutant and IDH-wild type tumors, adult-type diffuse gliomas are divided into distinct categories on the basis of IDH-mutation status in CNS 5, with the term glioblastoma, applying only to IDH wild-type tumors. IDH-mutant astrocytomas are classified separately and can encompass grade 2, 3, and 4 tumors. Adult tumors with both IDH mutations and 1q/19p co-deletion are classified as oligodendrogliomas, which can be grade 2 or 3, generally have a prolonged OS compared to tumors lacking these molecular changes (5).

In CNS 5, Pediatric-type diffuse gliomas are categorized into four distinct categories, with their histone mutation status playing a central role in their delineation: 1) Diffuse midline glioma, H3 K27-altered, 2) Diffuse hemispheric glioma, H3 G34-mutant, 3) Diffuse pediatric-type high-grade glioma, H3-wildtype and IDH-wildtype and 4) Infant-type hemispheric glioma (4).

For adults with HGG, standard of care employs the techniques of maximal surgical resection and radiation accompanied by chemotherapy followed by maintenance chemotherapy (6, 7). Most pediatric neuro-oncologists employ a similar treatment strategy for pHGG management for young patients diagnosed with hemispheric tumors who are old enough to receive radiation. Midline gliomas are often not amenable to resection due to their critical locations, and so only biopsy for diagnosis is performed prior to radiotherapy. Despite extensive clinical trials looking at various agents for the treatment of diffuse midline gliomas, thus far no additional therapy has proved to extend overall survival beyond radiation alone. For infant type hemispheric glioma, the concerns of damaging effects of irradiation on developing brains dictate a different treatment approach, to avoid radiation. Infant type hemispheric gliomas, also commonly have mutations which can be targeted with inhibitors, such as ALK, ROS, or NTRK alterations (8). With recent studies revealing deeper insights into gliomagenesis across all age groups and the development of cancer immunotherapy, the distinctions and commonalities of pHGG versus aHGG provide rationale for novel treatment options that are more specific and effective with potential to meaningfully improve the outcomes in patients with these tumor types.

## 2 Epidemiology

aHGG account for almost 25% of all CNS tumors in adults, with GBM being the most common type. In fact, GBM represents half of all malignant brain tumors diagnosed in adults; every year, there are over 10,000 new cases of GBM that are reported (9, 10). After diagnosis, GBM patients survive on average, 14.6 months and the 5-year survival rate is less than 5% (9, 11). Males are more often affected than females (1.6:1) and whites more often than blacks (2:1) (12, 13). The outcomes in patients over the age of 65 are worse when compared to those younger than 65 at the time of diagnosis (14). The outcomes may be worse because more elderly patients may not be able to tolerate standard of care therapies due to concern of toxicities and comorbid conditions.

When compared with adults, HGG in the pediatric population is less common and makes up only 3-15% primary central nervous system tumors in children (15). An overall survival of 10-73 months has been reported in pHGG. These wide ranges in reported epidemiologic data may be in part due to the changing classifications of HGG in the pediatric population over time, as well as differing inclusion criteria for HGG in pediatric clinical trials. Hemispheric HGG in the pediatric population is seen most frequently between the ages of 15 and nineteen (15), but in younger children, diffuse midline gliomas are recognized more commonly. Although it is unclear why, the disease has a predilection for males in both the pediatric and adult populations (15).

## 3 Clinical presentations

The challenge with diagnosing HGG includes the nonspecific nature of presenting symptoms. Both adult and pediatric patients can present with headaches and fatigue spanning a few months. In adults, the headache has been described to be similar to that of a tension headache or migraine (16). The headaches may be combined with cognitive deficit and mood changes. However, most patients are unlikely to see a primary care provider due to these symptoms. The most dramatic presentation of hemispheric HGG tends to be seizures. They can be seen at diagnosis in approximately 20% of adults and 30% of children (16, 17). Other symptoms that have been seen in children diagnosed with HGG are failure to thrive, lethargy and emesis, and cranial neuropathies which may be seen in patients with midline gliomas. Genetic predisposition syndromes such as Li-Fraumeni Syndrome, Tuberous Sclerosis, NF-1 and constitutional mismatch repair deficiency are associated with pediatric glioma patients (18–20).

## 4 Imaging and diagnosis

HGG is most commonly suspected based on imaging findings. Modalities include computed tomography and magnetic resonance imaging (MRI). On initial presentation, patients receive a computed tomography scan due to symptoms that could indicate stroke or other pathology within the brain. Once a bleed or mass in the brain is seen, a contrast enhanced MRI is performed.

In HGGs such as GBM, an infiltrative, heterogenous mass with edema and necrosis is usually seen. Imaging also focuses on areas of necrosis that may have some enhancement. Tumors such as GBM are vascular by nature and imaging is able to magnify this finding.

For hemispheric HGG, maximal safe surgical resection of the gross tumor is recommended. For tumors in midline or other eloquent locations, a biopsy may be obtained for tissue diagnosis. Depending on where the tumor is, there may not be enough tissue to justify the risk of obtaining a biopsy. This is especially true if HGG is located in the corpus callosum, the butterfly region of the brain that interconnects the two cerebral hemispheres. Imaging plays a critical role for surgical planning. Perfusion weighted imaging can characterize the tumor and aid in predicting the tumor grade and multimodal imaging allows the surgeon to determine the ideal target for a biopsy sample (21).

## 5 Molecular studies

Although aHGG and pHGG are histologically indistinguishable, the molecular biology of these tumors has shown significant differences in their genomic footprints. As underscored by the WHO CNS 5, IDH mutation status plays a key diagnostic and prognostic role in adult-type diffuse gliomas. IDH mutations confer an improved overall survival in adults with high grade gliomas when compared to those with IDH wild-type disease (22). However, with-in IDH mutated astrocytomas, the co-existence of CDK2A/B homozygous deletions are associated with poorer outcomes, and dictate an automatic grade 4 designation. For the IDH-wildtype tumors, several hallmark mutations correlated with poorer outcomes also automatically dictate a grade 4 tumor, regardless of the presence of characteristic histologic features such as microvascular proliferation or necrosis (8). These mutations include amplification of epidermal growth factor receptor (EGFR), TERT promotor mutations, and chromosome 7/10 copy number variants. EGFR encodes a tyrosine kinase receptor that is involved with cell proliferation, differentiation and cancer development (23). EGFR amplification is seen in up to 60% of adult glioblastomas while its occurrence in pediatric high grade tumors is less frequent (24). EGFR alterations represent an important therapeutic target for both small molecule inhibitors as well as immunotherapeutics. The PTEN gene is an important tumor suppressor gene that can inhibit cell invasion, prevent cell adherence to the matrix and formation of blood vessels. PTEN mutations are seen in a large proportion of aHGG patients and are rare to find in pHGGs (24, 25). TP53 is another important tumor suppressor gene, which is mutated in a subset of aHGG. The p53 pathway which also includes CDK2A, MDM2, in addition to TP53 is altered in the majority of adult GBM tumors.

In regards to pHGG, histone alterations play an important biologic and prognostic role. The majority of diffuse midline gliomas harbor a K27M mutation at histone 3.3 or histone 3.1. G34R or G34V mutations of H.3. are seen in hemispheric pHGG. Histone mutant tumors have a poorer OS than those that are wildtype. Given the prevalence of histone mutations in pediatric high grade gliomas, attempts to target these epigenetic pathways are underway (26). Like in aHGG, TP53 mutations are also common in pHGG, often found with co-existing histone mutations. These key molecular findings in aHGGs and pHGGs are summarized in **Table 1**.

**Table 1:** Molecular profiling of pediatric and adult high grade glioma.

Molecular Studies	pHGG	aHGG	Implications
IDH1	In COG ACNS0423, 16.3% (7/43 cases) of pediatric tumors and there was an age association that was apparent. Of those 43, 20 cases were in children >14 years and 35% of those cases (13) had an IDH1 mutation (Pollack IF 2011)	~50% of primary HGG cases (Haque A 2011)	Tumors with IDH mutations are classified as IDH mutant astrocytomas. The term glioblastoma no longer applies to IDH mutant tumors in WHO CNS5.
Overexpression of EGFR	0-80% of cases across studies (Suri V 2009)	Increased (Penas-Prado M 2012) EGFR amplification in 27%-60% cases (Haque A 2011)	Molecular characteristic of Grade 4 tumor
TERT promoter mutations	Rare (Koelsche C 2013)	40-70% of cases (You H 2017)	Molecular characteristic of Grade 4 tumor
Copy number alterations of chromosome 7 or 10	ND	Increased (Penas-Prado M 2012) 50-70% of cases (Haque A 2011)	Molecular characteristic of Grade 4 tumor
Mutation or loss of p53	33%-58% in pediatric cases (Suri V 2009)	Increased (Penas-Prado M 2012) 30% -60% of primary glioblastoma cases (Haque A 2011)	P53 is an important tumor suppressor
Loss or mutation of the PTEN gene	0%-20% of cases (Suri V 2009)	Increased (Penas-Prado M 2012) 27%-60% of cases (Haque A 2011)	PTEN is a tumor suppressor gene
H3 K27 mutant	60-80% of midline HGG (Graham MS 2020)	ND	Characteristic of pediatric diffuse midline gliomas
H3 G34 mutant	~20% of hemispheric HGG (Graham MS 2020)	ND	Characteristic of pediatric diffuse hemispheric gliomas

EGFR, epidermal growth factor receptor; pHGG, pediatric high grade glioma; aHGG, adult high grade glioma; PTEN, phosphate and tensin homolog; ND, not determined; MGMT, O6-methylguanine-DNA methyltransferase; MMR, mismatch repair; IDH, isocitrate dehydrogenase.

## 6 Treatment

### 6.1 Chemotherapy

Currently when patients are diagnosed with HGG, they are offered treatment with a goal to extend their lives as long-term survival from these tumors is exceedingly rare (16). Standard treatment offered at diagnosis for both the adult and pediatric populations diagnosed with HGG include surgical resection of maximal tissue with accompanying chemotherapy and radiation therapy. Temozolomide, an oral alkylating agent, is most commonly used for adjuvant chemotherapy. Stupp et al. showed that a HGG patients who received temozolomide concurrently with radiation had increased progression-free survival and overall survival when compared with those who did not (27). The two year survival in the cohort that received radiotherapy and temozolomide was 26.5%, with a median progression free survival of 6.9 months, compared to the group that only received radiotherapy whose two year survival was 10.4% with a median progression free survival of 5 months. However, apart from one study (28) most trials indicated that temozolomide chemotherapy had no effect on survival in children.

### 6.2 Molecular targeted therapy

Recently better understanding of the molecular biology of HGG have resulted in the development of a number of molecular targeted therapies with the hope of slowing the progression and ultimately developing a cure for HGG (29–31). These include therapies targeting tyrosine kinase receptors, tumor growth factor pathways, angiogenic pathways and intracellular signaling pathways.

EGFR and PDGFR (Platelet-Derived Growth Factor Receptor) amplification is commonly seen in aHGG (9). The kinase domain of EGFR and PDGFR and the downstream signaling can be selectively inhibited by tyrosine kinase inhibitors. However, Gefitinib and Erlotinib, oral EGFR inhibitors, and Imatinib, PDGFR inhibitor, have shown only limited benefit in clinical outcomes (9). The lack of expected success could be due to the redundancy of tumorigenic pathways in these tumors; blockade of a single pathway had limited effects.

Drugs targeting angiogenesis including bevacizumab, cedarinib, sunitinib and vatalanib have been investigated in patients with HGG as it is a highly vascularized tumor. However, pHGG patients were not as responsive to bevacizumab as the adult patients (32, 33). Combining irinotecan with bevacizumab (NCT00381797) has also not improved the prognosis in pediatric patients (34, 35).

Inhibiting the intracellular signaling pathways such as PI3K and mTOR as targeted therapy has also been evaluated in HGG. However, these have shown minimal efficacy in tumor control at tolerated doses (7). The redundant signaling pathways, drug toxicity, and blood brain barrier (BBB) are hurdles need to be overcome for further development of PI3K or PI3K/mTOR dual inhibitors in clinical trials (36).

### 6.3 Immunotherapy

The accelerated progress in the field of cancer immunotherapy has brought significant improvements of survival and quality of life for subsets of patients with cancer. In patients with the devastating HGG, harnessing the immune system to become more effective and specific with lower toxicity compared to current standard of care presents the hope of bringing a cure to the patients. However, the low mutational burden, the intra-tumoral heterogeneity, the BBB, and the immunosuppressive tumor microenvironment (TME), are all challenges for immunotherapy development in HGG. Nevertheless, a number of immunotherapeutic modalities are being tested in HGG preclinically and clinically (**Table 2**) and hold great promise in the future. For pediatric patients, immunotherapies have become viable therapeutic options, some are changing the course of the treatment for pHGG, while others need to be further evaluated.

**Table 2:** List of current immunotherapy clinical trials for adult and pediatric high grade gliomas. .

Immunotherapy	Trial ID	Phase	Patients	Sponsor/PI	Therapeutic Mechanism	Link
Vaccines	NCT01130077	1	P	Ian F. Pollack, MD	HLA-A2 restricted peptide vaccine with Poly-ICLC	https://clinicaltrials.gov/ct2/show/NCT01130077
	NCT01808820	1	P, A, OA	Macarena De la Fuente, MD	DCV	https://clinicaltrials.gov/ct2/show/NCT01808820
	UMIN000011030		P	Shinshu University Hospital	WT1 peptide vaccine	https://center6.umin.ac.jp/cgi-open-bin/ctr_e/ctr_view.cgi?recptno=R000012638
	NCT04280848	2	A,OA	Centre Hospitalier Universitaire de Besancon	UCPVax vaccine, TMZ	https://clinicaltrials.gov/ct2/show/NCT04280848
	NCT02454634	1	A,OA	National Center for Tumor Diseases, Heidelberg	IDH1 peptide vaccine	https://clinicaltrials.gov/ct2/show/NCT02454634
	NCT02455557	2	A,OA	Roswell Park Cancer Institute	SurVaxM, TMZ	https://clinicaltrials.gov/ct2/show/NCT02455557
	NCT02498665	1	A,OA	Sumitomo Pharma Oncology, Inc	DSP-7888	https://clinicaltrials.gov/ct2/show/NCT02498665
	NCT03665545	1/2	A,OA	University Hospital, Geneva	IMA950/Poly-ICLC, PEM	https://clinicaltrials.gov/ct2/show/NCT03665545
	NCT03299309	1	P,A	Eric Thompson, M.D.	PEP-CMV	https://clinicaltrials.gov/ct2/show/NCT03299309
	NCT04201873	1	A,OA	Jonsson Comprehensive Cancer Center	ATL-DC, PEM	https://clinicaltrials.gov/ct2/show/NCT04201873
Oncolytic virus	NCT02457845	1	P, A	U of Alabama at Birmingham	G207 and radiation	https://clinicaltrials.gov/ct2/show/NCT02457845
	NCT03896568	1	A, OA	M.D. Anderson Cancer Center	DNX-2401	https://clinicaltrials.gov/ct2/show/study/NCT03896568
	NCT04482933	2	P, A	U of Alabama at Birmingham	HSV-G207 with radiation	https://clinicaltrials.gov/ct2/show/NCT04482933
	NCT03178032	1	P	Clinica Universidad de Navarra	DNX-2401	https://clinicaltrials.gov/ct2/show/NCT03178032
ICI	NCT02359565	1	P, A	National Cancer Institute	Pembrolizumab	https://clinicaltrials.gov/ct2/show/NCT02359565
	NCT02667587	3	A, OA	Bristol-Myers Squibb	Niv, TMZ	https://clinicaltrials.gov/ct2/show/study/NCT02667587
	NCT02017717	3	P, A, OA	Bristol-Myers Squibb	Niv, Bev, Ipi	https://clinicaltrials.gov/ct2/show/NCT02017717
	NCT04323046	1	P, A	Sabine Mueller, MD, PhD	Niv, Ipi	https://clinicaltrials.gov/ct2/show/NCT04323046
	NCT02829931	1	A, OA	Moffitt Cancer Center	Niv, Ipi, Bev	https://www.clinicaltrials.gov/ct2/show/NCT02829931
Antibodies/ADC	NCT00445965	2	P, A, OA	Sloan Kettering Cancer Center	I131 3F8	https://clinicaltrials.gov/ct2/show/NCT00445965
	NCT03275402	2/3	P, A	Y-mAbs Therapeutics	131I-omburtamab	https://clinicaltrials.gov/ct2/show/NCT03275402
T/CAR T	NCT04099797	1	P, A	Baylor College of Medicine	C7R-GD2.CAR T	https://clinicaltrials.gov/ct2/show/NCT04099797
	NCT04185038	1	P, A	Seattle Children’s Hospital	B7H3-CAR T	https://clinicaltrials.gov/ct2/show/NCT04185038
	NCT04077866	1/2	A, OA	Zhejiang University Hospital	B7H3-CAR T	https://clinicaltrials.gov/ct2/show/NCT04077866
	NCT01082926	1	A,OA	City of Hope Medical Center	IL13Rα2 CAR T	https://www.clinicaltrials.gov/ct2/show/NCT01082926
	NCT04045847	1	A, OA	Xijing Hospital	CD147 CAT T	https://clinicaltrials.gov/ct2/show/NCT04045847
NK/CAR NK	NCT05108012	1	P, A	Royan Institute	Ex Vivo Activated Haplo-identical NK	https://clinicaltrials.gov/ct2/show/NCT05108012
	NCT03383978	1	A, OA	Johann Wolfgang Goethe University Hospital	NK-92/5.28.z Cells	https://clinicaltrials.gov/ct2/show/NCT03383978
**Table 2** Continued						
Combinatorial immunotherapy	NCT04808245	1	A, OA	German Cancer Research Center	H3K27M peptide vaccine and Atezolizumab	https://www.clinicaltrials.gov/ct2/show/NCT04808245
	NCT03879512	1/2	P, A	Wuerzburg University Hospital	DCV, depleted regulatory T-cells & Niv/Ipi	https://clinicaltrials.gov/ct2/show/study/NCT03879512
	NCT03334305	1	P, A	University of Florida	TMZ, vaccines, GM-CSF, Autologous HSCs	https://clinicaltrials.gov/ct2/show/NCT03334305
	NCT02960230	1/2	P, A	Sabine Mueller, MD, PhD	K27M peptide vaccine, niv	https://clinicaltrials.gov/ct2/show/NCT03334305

P, pediatric; A, adult; OA, older adult; ICI, immune checkpoint inhibitor; HLA, human leucocyte antigen; DCV, dendritic cell vaccine; WT1, wilms tumor gene; BM-hMSC, bone marrow derived human mesenchymal stem cells; PEM, pembrolizumab; HSV, herpes simplex virus; Niv, nivolumab; Bev, bevacizumab; Ipi, ipilimumab; ADC, antibody drug conjugate; CAR, chimeric antigen receptor; NK, natural killer; TMZ, temozolomide; GM-CSF, granulocyte-macrophage colony-stimulating factor.

#### 6.3.1 Vaccines

**Figure 1:**
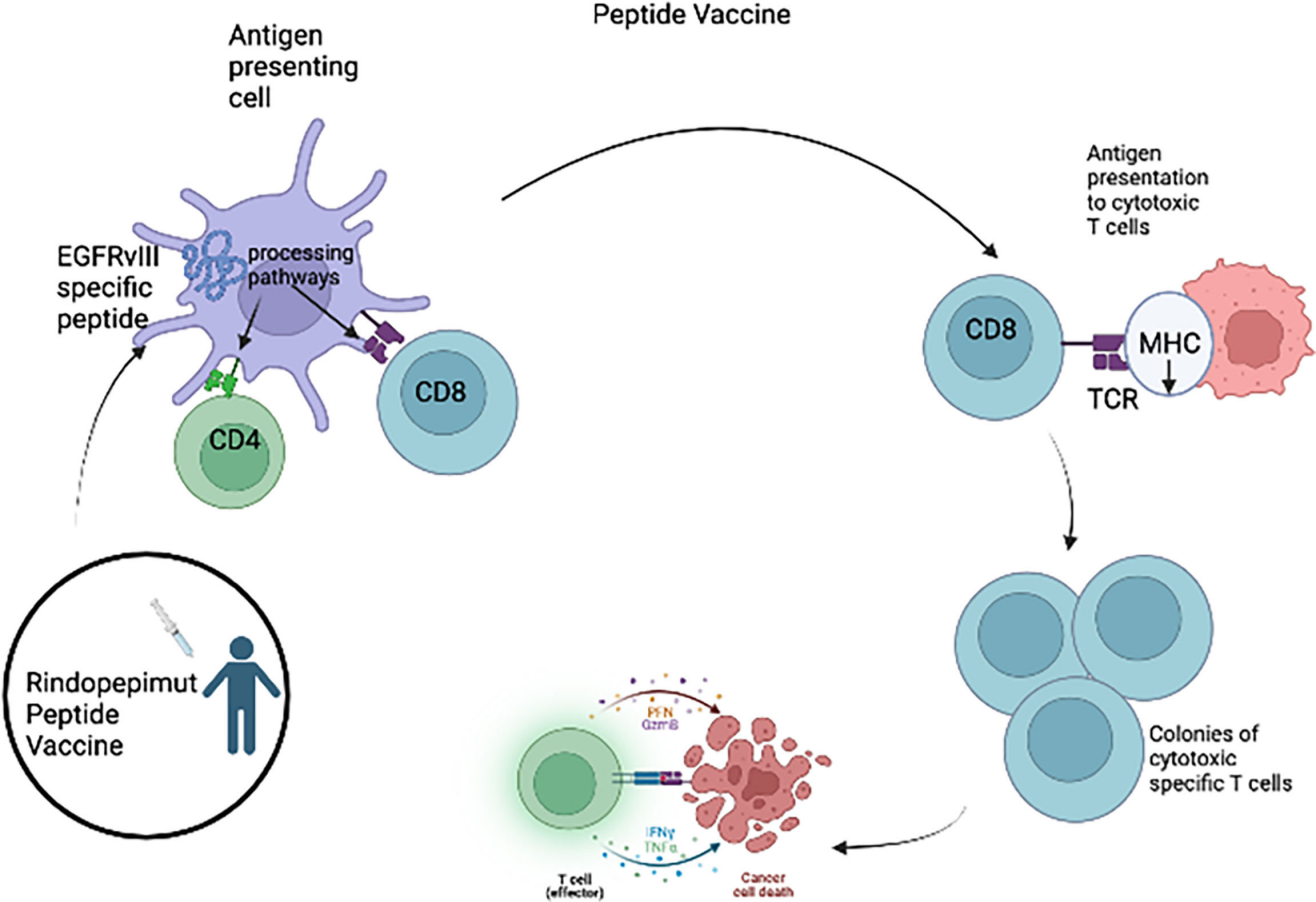
For the Rindopepimut vaccine, EGFRvIII specific peptide is introduced into the bloodstream and the peptide is processed and presented by the APC causing the activation of CD4 helper T cells and CD8+ cytotoxic T cells. The cytotoxic T-cells are then able to cause lysis of the glioma tumor cells.

One type of immunotherapy utilized in HGG is cancer vaccines (**Figure 1**), which rely on class I presentation of tumor associated antigens. HGG-associated antigens in a vaccination were administered to patients to stimulate an immune response. These antigens are usually small peptides that activate the cytotoxic T lymphocytes (CTLs). Rindopepimut is an injectable peptide which stimulates a response against a specific EGFRvIII antigen, which is expressed in HGG. Clinical trials of adult patients with HGG expressing EGFRvIII have shown positive results with rindopepimut (9, 37–39). However, a recent trial of rindopepimut/temozolomide in patients harboring the EGFRvIII mutation showed disappointing results (40). No significant difference was observed in overall survival in the rindopepimut cohort (20.1 months) compared to the placebo (20.0 months).Other tumor specific antigens for aHGG vaccines include TERT, IDH1, survivin and WT1. Clinical trials evaluating peptide vaccines targeting these antigens in adult GBM patients are ongoing (NCT04280848, NCT02454634, NCT02455557, NCT02498665, NCT03665545) (**Table 2**). Early results have shown that these vaccines are safe and immunogenic (41–44).

Cytomegalovirus (CMV) infects most adults and CMV phosphoprotein 65 (pp65) expresses on greater than 50% of GBM but not on normal brain parenchyma (45). A phase I trial called PRiME (NCT03299309) (**Table 2**) is ongoing to test pp65 peptide vaccine in both adult and pediatric patients with malignant glioma.

In pediatric glioma patients, peptide vaccines targeting tumor-associated antigens including ephrin type-A receptor 2 (EphA2), interleukin-13 receptor alpha 2 (IL13Rα2), survivin and WT1, have been developed and tested (NCT01130077, UMIN000011030) (**Table 2**). The vaccination was generally well-tolerated in children and induced modest immune and clinical responses (46–50). An early phase vaccine trial utilizing the immunogenic peptide of the H3K27M mutation (51) in pediatric patients with midline gliomas harboring this mutation is also ongoing (NCT04808245) (**Table 2**).

**Figure 2:**
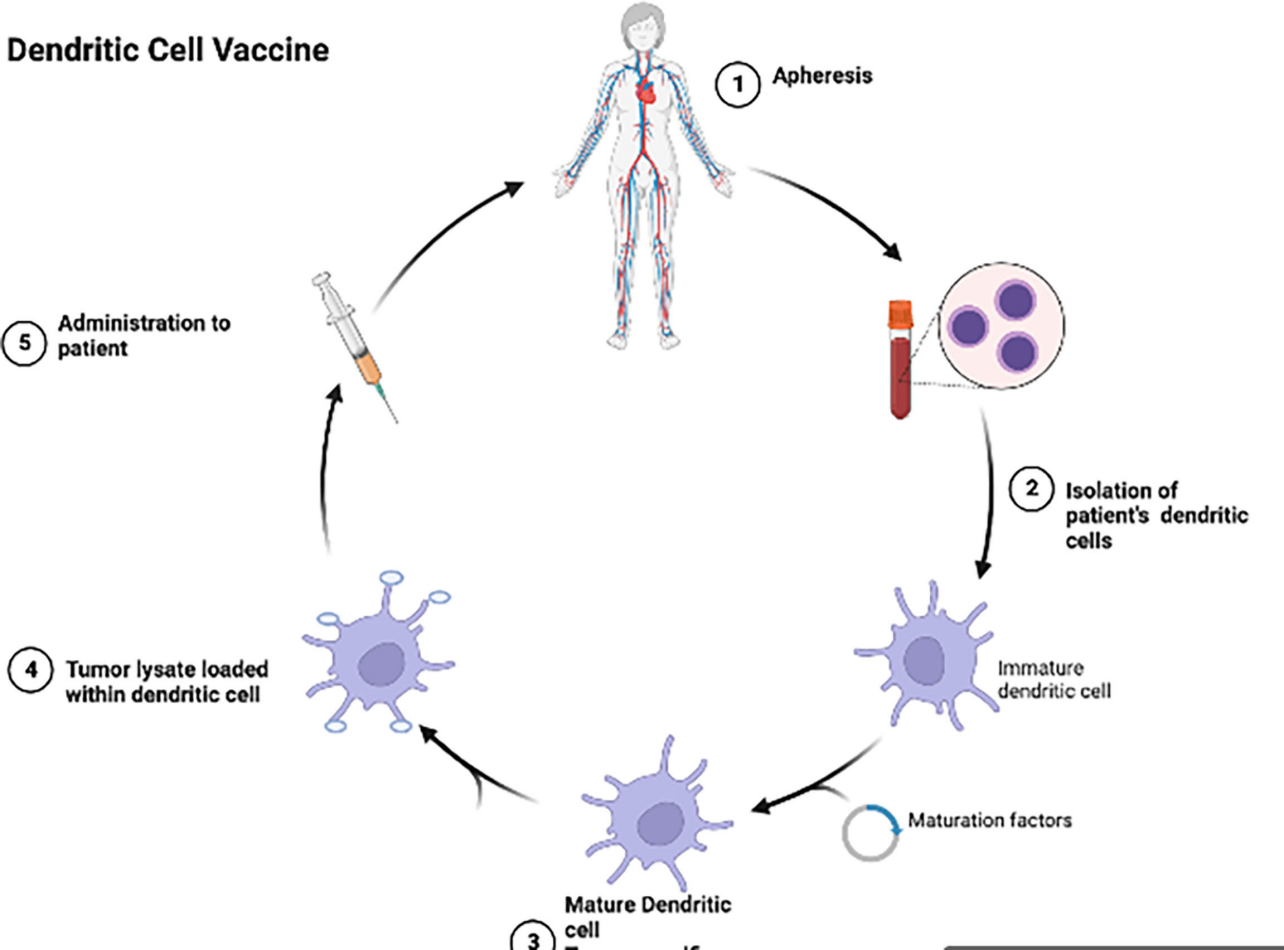
Dendritic cells are isolated from the patient’s blood, allowed to mature and then tumor lysate is loaded into the dendritic cells and given back to the patient as a vaccine.

Dendritic cell vaccines (DCV) (**Figure 2**) have been investigated in multiple trials in aHGG patients (52). Dendritic cells loaded with glioma antigens or whole tumor lysate are administered to patients for T cell activation and cytotoxic activity. Early phase clinical trials of DCs pulsed with single antigen including EGFRvIII, WT1, CD133 or IL13Rα2 in adult GBM patients demonstrated these DCVs to be immunogenic with no serious adverse events (38, 53, 54). However, ICT-107, a multi-peptide pulsed DCV which consists of autologous dendritic cells pulsed with six synthetic peptides: melanoma-associated antigen-1 (MAGE-1), antigen isolated from immunoselected melanoma-2 (AIM-2), Her2/neu, tyrosine-related protein-2 (TRP-2), glycoprotein 100 (gp100), and IL-13Rα2 (55), has shown no significant difference in overall survival in the treatment group as compared to controls in a randomized phase II trial in newly diagnosed adult GBM patients (55). Tumor lysate pulsed DCVs in aHGG patients have also shown mixed results. DC-VaxL is under phase III trial and exciting interim results have shown median overall survival of 23.1 months from surgery for the intent-to-treat population (56). However, another tumor lysate pulsed DCV (Audencel) evaluated in a randomized, controlled phase II study of patients with newly diagnosed GBM showed no significant difference in overall survival between the treatment and control groups (57). A phase I trial involving an autologous tumor lysate pulsed DCV (ATL-DC) and pembrolizumab in recurrent adult GBM is currently ongoing (NCT04201873).

DCVs have now been moved into the pediatric population. In the HGG-IMMUNO trial, De Vleeschouwer and colleagues evaluated tumor lysate DCV in children and adults with relapsed HGG (58). Improved progression-free survival was seen in the cohort receiving the tumor lysate DCV. It should be noted that younger age was correlated with improved overall survival, although it is unclear whether the difference in survival is caused by different underlying biology and natural course of the tumors in different age groups. A more recent report on DCVs with pulsed tumor lysate in a cohort of pediatric HGG patients with a small sample size also showed promising results (59). Three patients received DC vaccinations and of those two were alive 51 and 40 months from the time of enrollment into the study. The first patient was described to possibly have had a partial response on MRI after dendritic cell vaccination while the second patient was attending school at an age appropriate level and the third patient suffered from mild hemiparesis but was otherwise thriving and successful cognitively. Currently, a number of DCV trials are open and recruiting pHGG and aHGG patients (60) (**Table 2**).

Although dendritic cell vaccinations are currently in trials, one challenge with this therapeutic modality is HLA downregulation. HLA1 and HLA2 are the most common antigens utilized for cancer vaccines and downregulation or loss of HLA class 1 molecules can prevent tumor cells from being recognized. Zagzag et al. noted that downregulation of major histocompatibility complex (MHC) class I and II antigens allow for the migration and invasion of glioma cells. This downregulation allows for the tumor to go undetected by the immune system and can also explain, in part, the lack of inflammatory infiltrate surrounding glioma cells (61).

#### 6.3.2 Oncolytic virus

Oncolytic viruses have shown promising efficacy in treating cancer (62, 63). They mediate antitumor effects by directly lysing cancer cells while sparing normal cells as well as stimulating immune response and modulating tumor microenvironment towards less immunosuppressive phenotype. Since the first oncolytic viruses -based immunotherapy (T-VEC) (64) has been approved by FDA for treating melanoma patients in 2015, oncolytic viral therapy for other tumor types have also developed to various extent (63). These agents have a particular attraction for brain tumors as they are generally injected intratumorally and thus best for localized disease.

Over the past three decades, various families of viruses, including herpes simplex virus type 1, adenovirus, Newcastle disease virus, reovirus, vaccinia virus and others, have been assessed in preclinical studies for their potential value in the treatment of HGG (65). In a case study in Germany, four aHGG patients were treated with three viruses: Newcastle disease virus, parvovirus, and vaccinia virus. This oncolytic virotherapy was well-tolerated by the patients and induced complete response or stable disease in these patients with overall survival ranging from 4 to 14 years. Importantly, the oncolytic virotherapy improved quality of life of the patients for years after the treatment (66). Multiple early-stage oncolytic viral therapy clinical trials in aHGG patients are currently active in the United States (67). In 2021, Delytact (teserpaturev/G47∆) was approved in Japan as the first oncolytic viral therapy for adult malignant glioma in the world based on the result of a phase II single arm trial showing a survival benefit and good safety profile of G47Δ in patients with residual or recurrent HGG after chemotherapy and radiation therapy (UMIN000015995) (68). In this trial, up to six doses of G47Δ were intratumorally administered in 19 adult patients. The survival rate one year after G47Δ initiation was 84.2%, the median overall survival was 20.2 months after G47Δ initiation and 28.8 months from the initial surgery (68).

Recently, Ring and colleagues reported that compared to aHGG xenografts, patient-derived pHGG xenografts were more sensitive to G207, a genetically engineered herpes simplex type 1 virus (HSV-1) (69). The favorable safety profile and efficacy of G207 in the adult clinical trials resulted in the design of a phase I clinical trial of oncolytic viral therapy with G207 in pediatric patients with HGG. The results of this trial showed acceptable adverse-event profile and evidence of radiographic, neuropathological or clinical responses (70).

Delta-24-RGD is an adenovirus engineered to be competent in replication in tumor cells with a defective RB signaling pathway. It was reported that Delta-24-RGD elicited an antitumor effect and prolonged animal survival in pediatric glioma and diffuse intrinsic pontine glioma (DIPG) mouse models (71). These data, and the proven safety and effectiveness of this virus in adult gliomas, have led to a phase I/II clinical trial of DNX-2401 oncolytic viral therapy in pediatric patients with newly diagnosed DIPG (NCT03178032). In this study, 12 patients between ages 3-12 were enrolled and they received a single infusion of DNX-2401 and subsequent radiotherapy was received by 11 patients. Over a median follow up of 18 months, a reduction in tumor size was assessed *via* MRI in nine patients; partial response was observed in three patients and stable disease in the other eight; median progression free survival and overall survival were 10.7 and 17.8 months, respectively (72).

#### 6.3.3 Immune checkpoint blockade

Immune checkpoint blockade (ICB), antibody therapy directed against several negative immunologic regulators, cytotoxic T lymphocyte–associated antigen 4 (CTLA-4) and the programmed cell death protein 1 pathway (PD-1/PD-L1), have shown significant success in various malignancies. ICBs such as Ipilimumab (anti-CTLA-4) and pembrolizumab (anti-PD-1), are approved by the FDA for the treatment of a wide spectrum of cancers (73). In aHGG, phase I of the CheckMate 143 trial demonstrated safety and tolerability of nivolumab (anti-PD-1) and ipilumumab (74). However, no single agent therapy demonstrated a survival benefit, including bevacizumab and nivolumab, in patients with recurrent HGG in the phase III trial (75). A number of clinical trials in adults are ongoing to investigate other combinations of ICBs alone and in combination with radiotherapy (**Table 2**).

Utilization of biomarkers to stratify and identify potentially responsive patients is an important consideration in ICB immunotherapy. PD-L1 expression has been reported to correlate with response to ICBs in adult cancer. Higher level of tumor mutational burden is also associated with an increased expression of neoantigens and enhanced efficacy of ICBs. Interestingly, compared to adult counterparts, pediatric gliomas have been found to have higher levels of microsatellite instability (MSI), a surrogate biomarker for tumor mutational burden (76). Boefett et al. treated two siblings with relapsed HGG with MSI using nivolumab and observed clinically significant responses and a profound radiologic response (77). However, the success of nivolumab was not repeated in another pediatric relapsed HGG case with MSI (78). Several large studies are ongoing to evaluate the efficacy and safety of ICBs in pHGG in a more rigorous manner (**Table 2**). As with vaccines, ICB rely on expression of tumor associated antigens, which can escape detection with the down regulation of HLA class I expression.

#### 6.3.4 Antibody and antibody drug conjugates (ADC)

**Figure 3:**
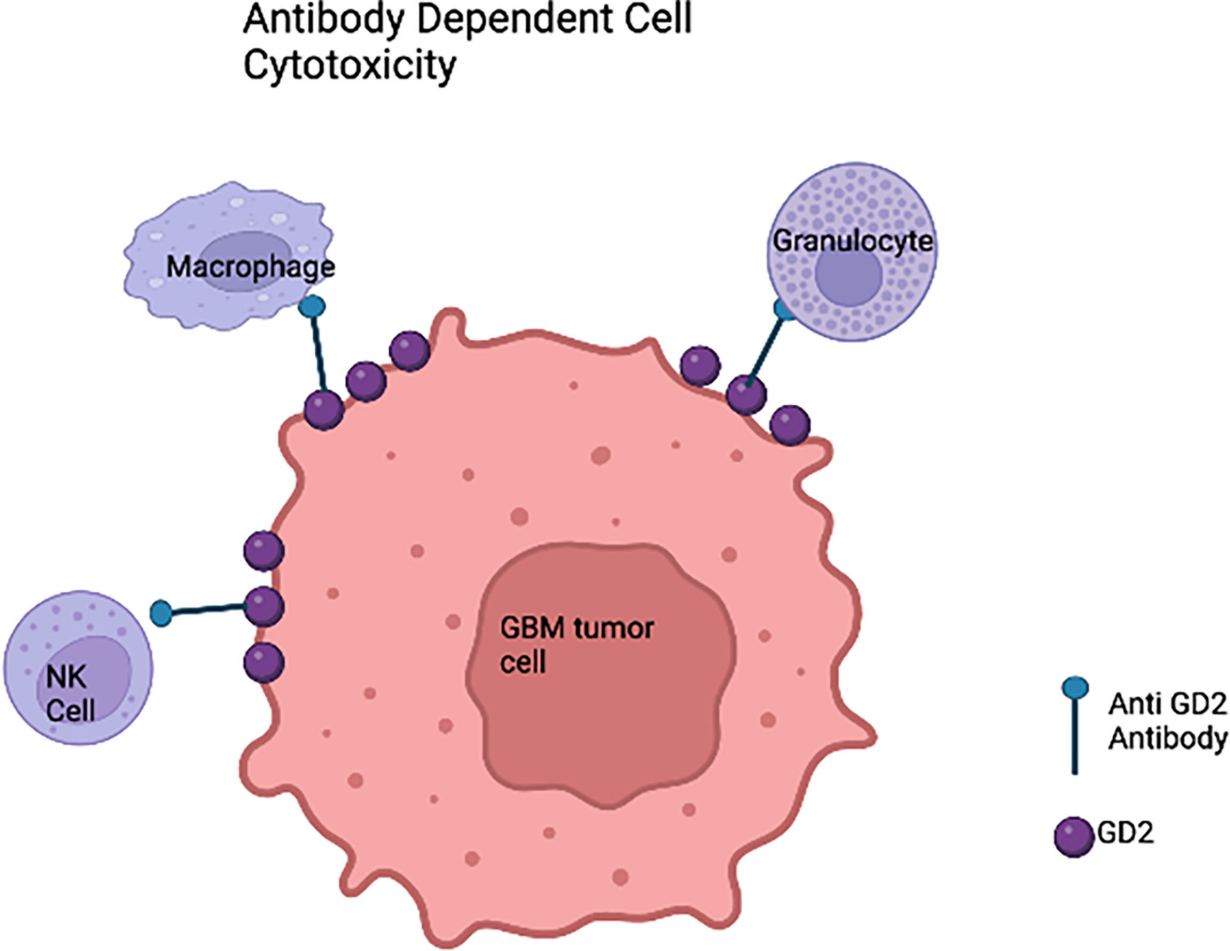
Anti-GD2 immunotherapy of glioblastoma cells based on the innate immune system. In the presence of anti-GD2 monoclonal antibodies,GBM cells would be susceptible to the activity of natural killer (NK) cells that would mediate antibody-dependent cell mediated cytotoxicity (ADCC), granulocyte mediated ADCC, and monocyte macrophage ADCC.

Recent progress and efficacy of antibody-based immunotherapy (**Figure 3**) have promoted the searching for clinically relevant tumor antigens as immunotherapy targets in solid tumors. Gangliosides are sphingolipids that contain carbohydrates and are widely expressed in normal tissues, which renders most of them ineffective for cancer therapy. However, the disialoganglioside GD2 subtype is overexpressed in many tumors with minimal expression in normal tissues (79). Over the past few decades, several anti-GD2 monoclonal antibodies (mAbs) have been developed (80–83). Anti-GD2 mAbs target GD2 expressing tumor cells and recruit Fc-receptor-expressing innate immune cells such as natural killer (NK) cells and macrophages to mediate antibody dependent cell mediated cytotoxicity (ADCC) and/or phagocytosis (12). Indeed, anti-GD2 mAbs significantly increased survival rates and transformed the landscape for pediatric patients with primary and relapsed neuroblastoma (84–87). It is also well known that GD2 is expressed on HGG cells. A chimeric anti-GD2 antibody dinutuximab beta induces ADCC and has anti-tumor efficacy against HGG cells. This study provided rationale for GD2 directed immunotherapy against HGG (88). A phase II study of intrathecal I-3F8 anti-GD2 mAb in patients with GD2 expressing CNS neoplasms in all age groups is ongoing (NCT00445965).

B7-H3, also called CD276 or B7RP-2, belongs to the B7 ligand family. B7-H3 is highly expressed on differentiated tumor cells, metastasis-initiating cells, tumor-associated vasculature and stroma, and has a limited distribution in normal tissues, all which features a favorable target for antibody-based immunotherapy (89). B7-H3 blocking mAbs have demonstrated efficacy in increasing tumor infiltration of CD8+ T cell and NK cell, limiting tumor growth, and/or improving animal survival in mouse models of various types of cancer including melanoma, colorectal cancer and ovarian cancer (90–92). In addition, B7-H3 mAbs have been used as vehicles to selectively deliver radioisotopes to B7-H3+ tumors in clinical trials (NCT03275402, NCT00445965). Convection-enhanced brainstem delivery of 124I-8H9 to DIPG tumors in patients of 2-21 years old has demonstrated to be safe with minimal systemic exposure of radioisotopes and no toxicity (NCT01502917) (93).

Another antibody based immunotherapy with high potential is antibody drug conjugates (ADC), which are composed of a mAb which specifically recognizes a surface protein and a cytotoxic payload (94). Unlike systemically administrated immunotherapy, ADCs directly and specifically deliver the cytotoxic payload to the tumor. This significantly decreases the toxicity of ADC. Furthermore, the additive/synergistic effect of the combination of the antibody and the payload significantly enhances tumor cell killing (95). Phase I and III clinical trials using EGFRvIII ADCs AMG 595 (NCT01475006) and ABT 414 (NCT02573324, NCT02343406), respectively, have shown favorable pharmacokinetics with possible benefit for adult patients with EGFRvIII-mutated HGG (96–98).

#### 6.3.5 Cytotoxic T lymphocytes and chimeric antigen receptor (CAR) T cell therapy

Adoptive cell transfer is also being studied as a novel therapeutic strategy in HGG. Autologous immune cells, mainly CTLs are isolated from peripheral blood mononuclear cells and activated ex vivo with autologous tumor cells (9).

**Figure 4:**
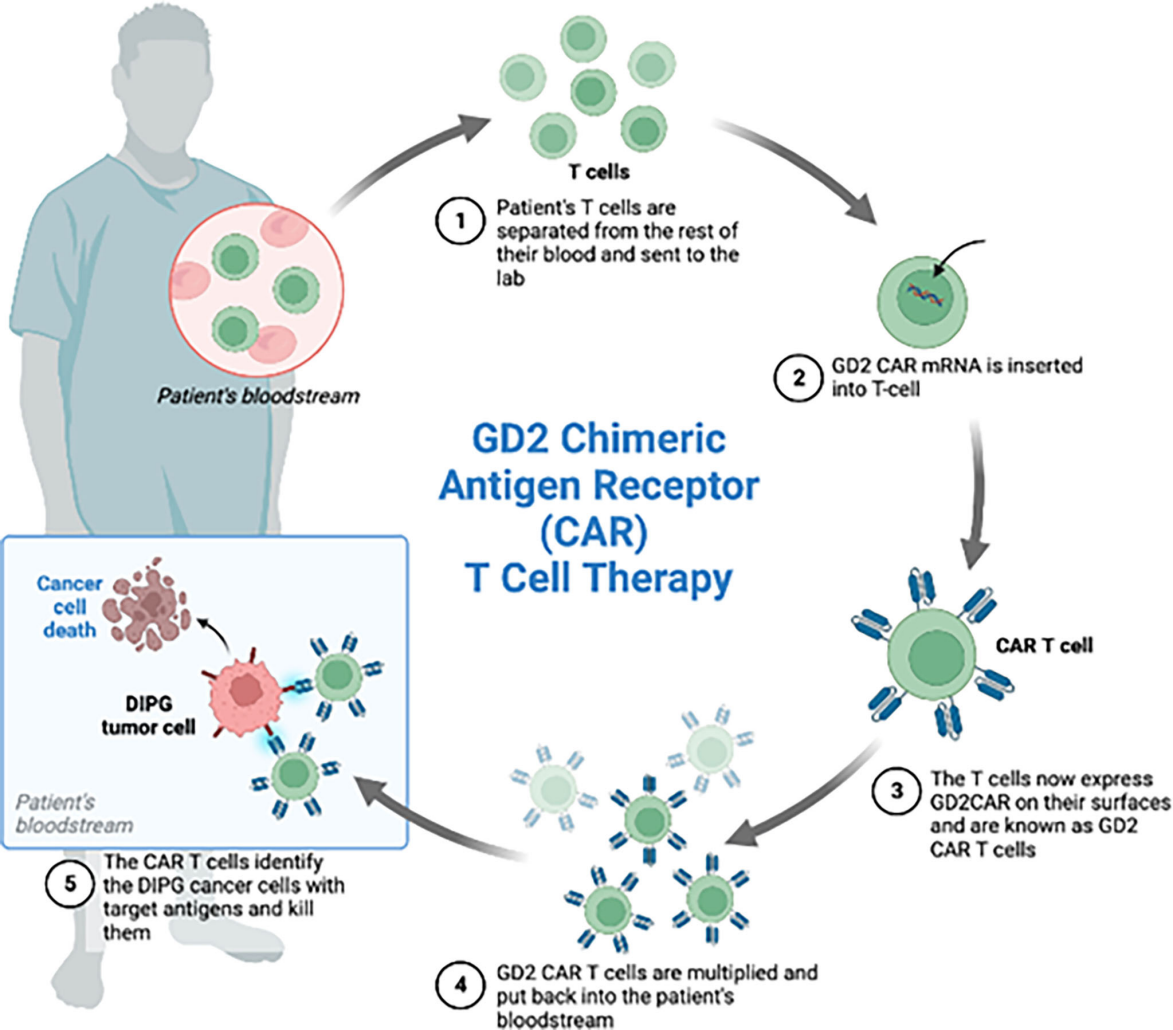
Four patients with the diagnosis of diffuse intrinsic pontine glioma were enrolled in the phase 1 clinical trial of GD2-CAR T cells. Each patient received GD2 CAR T cells. These CAR T cells were generated by isolating the patient’s T-cells from the rest of the blood, inserting GD2 CAR mRNA and administering the GD2 CAR T cells back to the patients. Expansion of CAR T cells, increased inflammation after treatment and increased CAR T cells in the CSF during peak inflammation was observed.

With respect to specificity and independence of MHC molecules, CAR represents a promising approach (**Figure 4**). A single chain variable fragment of an antibody is connected to the CD3 T cell receptor signaling domain and this helps target the T cell cytolytic activity. The target antigen can be engaged without presentation by the MHC (99). The FDA had originally approved CAR T cells for hematologic malignancies but many studies have shown potential efficacy of CAR T cells for brain tumors such as HGG, medulloblastoma, and ependymomas (11, 100).

In a pilot first-in-human study in adult patients with recurrent GBM, intracranial infusions of IL13Rα2 CAR T cells have shown excellent efficacy and no severe side effects (NCT01082926) (101). EGFRvIII targeting CAR T cells demonstrated effective control of xenograft tumor growth in a GBM preclinical mouse model (102). However, in a pilot trial in adult patients with GBM, adoptive transfer of EGFRvIII-CAR T cells did not demonstrate clinically meaningful effects (103). The limited efficacy of EGFRvIII CAR T cell therapy is in part due to the heterogeneous expression of EGFRvIII in glioma cells and the immunosuppressive TME in GBM (104, 105). CD147 is a transmembrane glycoprotein that belongs to the immunoglobulin superfamily. CD147 is overexpressed in common tumors including gliomas (106) and is associated with poor prognosis in GBM patients (107, 108). In adult patients with recurrent GBM, an early phase clinical trial of CD147-CAR T cells is currently in progress (NCT04045847).

A recent publication demonstrated the potential efficacies of CAR-T cells targeting pediatric diffuse midline gliomas (DMG). GD2 has been investigated in clinical and preclinical trials for neuroblastoma and GD2-CAR T cell therapies have been well tolerated in neuroblastoma (99). High GD2 expression level is correlated with the H3K27M mutation. In the patient derived H3K27M+ DMG orthotopic xenograft models, almost complete clearance of the tumor and improved animal survival were observed after the peripheral administration of GD2 specific CAR T cells (109). Mount et al. studied GD2 directed killing while using patient derived H3K27M-mutant glioma cell cultures. The group generated GD2-targeting CAR T cells and found significant GD2 dependent killing and cytokine release upon exposing to the CAR T cells targeting GD2 but not those targeting CD19 (110). Majzner and Ramakrishna et al. conducted the first Phase I dose escalation trial of GD2 CAR T cells in four children diagnosed with H3K27M+DMG. They found an increased concentration of CAR T cells in the tumor and clinical as well as radiological improvement in patients. Tumor inflammation associated neurotoxicity was the biggest concern in the trial as the inflammation of the brainstem transiently worsened with CAR T cell infusions in each of the patients (11).

B7-H3-CAR T cells with various B7-H3 antibody single chain variable fragments have been created and demonstrated potent *in vitro* cytotoxicity against HGG and effectively controlled tumor growth and prolonged animal survival *in vivo* (111, 112). Currently, B7-H3 CAR T cells are under evaluation in clinical trials in adult (NCT04077866) and pediatric patients with HGG (NCT04185038). A phase I trial of C7R-GD2 CAR T cells, which is engineered to express an interleukin-7 receptor (C7R) for constitutive activation of CAR T cells, is ongoing in both adult and pediatric patients with GD2-expressing HGG (NCT04099797).

#### 6.3.6 NK and CAR NK cell therapy

NK cells are as important in initiating cytotoxic activity against malignant tumor cells as CTLs. Unlike T cells, NK cells require no prior sensitization for tumor immunity. Their function and activity is regulated by various NK receptors. One class includes killer inhibitory receptors (KIRs) while the other receptors allow for the activation of NK cells. KIRs are recognized and bound by MHC class I molecules (MHC-I) on tumor cells which render the NK cells ineffective as they become inactivated. NK cells have also been shown to interact with DCs and play an integral role in the immune response along with DCs and CTLs. Administration of *in-vitro*-activated NK cells as adoptive cellular immunotherapy has shown efficacy in treating malignant tumors (25).

In glioma, very few NK cells infiltrate into the tumor. According to Kmiecik et al. (113), NK cells made up ~2% of all infiltrating immune cells and most of these NKs were CD56^dim^CD16^neg^ subtype. This phenotype showed activation in other tumors, suggesting although the abundance of NK cells in the glioma microenvironment is low, they have cytotoxic potential. Analysis of the HGG cell surface molecules showed the presence of high levels of MHC-I that interact with the NK inhibitory receptors. Blocking the interaction between the MHC molecules on HGG cells and KIRs on NK cells may aid in bringing more NK cells into the tumor microenvironment (TME) (114). Induction of NKG2D ligand expression by HDACi is another approach to overcoming immunosuppression to NK cells (115) as previously reported by our group and others (116).

Ishikawa et al. achieved decreased tumor volume without neurogenic toxicity in adult malignant brain tumor patients using autologous NK cell therapy. In 56% of the cases they studied, tumor size was reduced. In 19% of the cases, there was a significant reduction of the tumor size compared to what it was during the pretreatment phase (25).

Recent development of CAR NK cell therapy as a novel immunotherapeutic modality has been successful and addressed issues inherent to CAR T cell therapy such as side effects including cytokine release syndrome (CRS) and encephalopathies (ICANS). In Germany, a clinical trial is ongoing with HER2-CAR-NK-92 cells in adult patients suffering from recurrent HER2+ HGG (CAR2BRAIN, NCT03383978) (115). The recently completed dose-escalation study has shown no dose-limiting toxicities at neither of the three applied dose levels. Currently, patients are being recruited for the expansion cohort of the trial.

### 6.4 Combinatorial therapy

The poor prognosis of HGG is in part attributed to the highly heterogenous, metastatic and angiogenic nature of the tumor, leading to the tumor resistance to therapy. Combinatorial therapy approaches are being developed and tested in preclinical and clinical settings against HGG to overcome therapy resistance.

Temozolomide is the standard chemotherapy for HGG and has been tested extensively in combination with various regimens including cilengitide, bevacizumab, paclitaxel, irinotecan and tarceva preclinically (117–124) and in clinical trials in adult patients (18 years and older) (NCT00686725, NCT00689221, NCT00943826, NCT00262730, NCT00544817, NCT01402063, NCT00805961, NCT00979017, NCT00525525).

In a study on phosphor-tyrosine-mediated signaling pathways activated by EGFRvIII, the c-Met receptor tyrosine kinase was found to be EGFRvIII-cross-activated. This provided a rationale of a combinatorial therapy with an inhibitor of c-Met and an inhibitor of EGFR. The combination had an additive/synergistic anti-tumor effect demonstrated by enhanced cytotoxicity against EGFRvIII+ HGG cells compared to either compound alone, suggesting potential clinical value of the combination of c-Met and EGFR inhibitors in both adult and pediatric EGFRvIII+ HGG patients (125). In aHGG, a study conducted by Montano et al. identified 44% (32 of 73) of aHGG cases as EGFRvIII positive (126). In pHGG, Bax and colleagues found that only 17% (6 of 35) of pHGG cases they studied harbored EGFRvIII mutations (127). However, Li et al. found 44% (4 of 9) of pediatric DIPG cases to be EGFRvIII positive (128). More studies with larger sample size in both aHGG and pHGG are needed to determine if there is a significant difference in incidence of EGFRvIII in these age groups to contribute to a significant difference in response to the combination of c-Met kinase and EGFR inhibitors.

The combination of DC vaccines and ICB has shown effectiveness in the treatment of melanoma patients (129). Human cytomegalovirus (CMV) proteins and nucleic acids were found in the majority of aHGG. In a cohort of 25 pHGGs, approximately 67% of the samples express CMV antigen pp65 or IE1-72 as detected by immunohistochemical staining and *in situ* hybridization (130). With the hypothesis that nivolumab-induced ICB could increase effectiveness of DC vaccines in HGG, an early phase, single-center, randomized clinical trial to assess the safety of nivolumab combined with CMV pp65 mRNA pulsed DC vaccine in adult patients with recurrent resectable HGG was completed (NCT02529072). Safety of the combination in recurrent HGG patients is at similar level to that of nivolumab alone. However, due to lack of efficacy in improving overall survival in recurrent HGG patients shown in the CheckMate 143 trial, this study was terminated early. Continued evaluation of combination of new IBCs with DC or peptide vaccination in aHGG and pHGG is underway (**Table 2**) (NCT04808245, NCT03879512, NCT03334305, NCT02960230).

Myeloid-derived suppressor cells (MDSCs) are a very heterogeneous population of bone marrow derived immature myeloid cells. Under normal conditions, these immature myeloid cells have the potential to differentiate into multiple types of cells including macrophages, granulocytes, and DCs. In pathological states of HGG, the subverted differentiation of immature myeloid cells leads to the generation, expansion, activation and recruitment of MDSCs in the tumor bed as well as the peripheral blood (131, 132). MDSCs are present at very low frequencies in healthy human body. In adult patients with HGG, MDSCs constitute greater than 40% of the tumor-infiltrating immune cells and confer immunosuppression by expressing IL-4Rα, inducible nitric oxide synthase (iNOS), arginase, PD-L1, and CD80, and suppressing antigen-specific T cells (133, 134). MDSC depletion or checkpoint blockade was found to enhance the efficacy of HSV-1 thymidine kinase (HSV-1TK) and Fms-like tyrosine kinase ligand (Flt3L) gene therapy in aHGG (134). Only few studies investigated the role of MDSCs specifically in pHGGs. It was reported that high levels of circulating MDSCs correlate with poor prognosis in DIPG patients, indicating a role of MDSCs in immunosuppression and tumor immune evasion mechanism in pHGG (135) and a potential value of combinatorial immunotherapy targeting MDSCs in pHGG.

HGG resistance to NK cell therapy is in large part due to the small number of activated NK cells, poor NK *in vivo* persistence, and lack of specific tumor targeting (136). To overcome HGG NK resistance, our group combined approaches to increasing NK cell number and activity (ex vivo NK expansion), improving persistence and trafficking (IL-15 superagonist N-803), and enhancing tumor targeting (anti-GD2 mAb dinutuximab). N-803 combined with dinutuximab and ex vivo expanded NK cells significantly prolonged animal survival of GD2+ HGG xenografted NSG mice, providing the rationale for clinical investigation of this combinatorial NK cell therapy in patients with GD2+ HGG (137) as reported by our group.

HGG resistance to NK therapy is also due to an unfavorable and hypoxic TME that suppresses NK cell effector function (138). CD73 is a hypoxic ectoenzyme that drives the accumulation of adenosine, leading to significant impairment of NK cell activity (139). Wang et al. engineered anti-GD2-NKG2D bispecific CAR NK cells to simultaneously harbor a functional domain to inhibit the activity of CD73 independently of CAR to enhance NK cell activity. Compared to mock NK cells, these multifunctional engineered NK cells more potently targeted patient-derived HGG cells and tumors *in vitro* and *in vivo* (140). Since GD2 and CD73 are widely expressed on various lines of HGG cells, both from pediatric and adult patients (140), the above mentioned two combinatorial CAR NK cell therapy have potential generalizable clinical value in both pHGG and aHGG.

Oncolytic viruses can be armed with immunostimulatory molecules to enhance their immune-activating characteristics and are highly synergistic when combined with other immunotherapies such as NK cell therapy. In an immunocompetent model of high grade glioma, a virus expressing IL15/IL15Rα combined with off-the-shelf anti-EGFR CAR NK cells have shown synergistic effects on reducing tumor growth and extending animal survival in comparison to single agents by increasing NK and CD8+ T cells intracranial infiltration and activation and improving CAR NK cell persistence (141).

### 6.5 Barriers to treatment

Although immunotherapy is an attractive therapeutic strategy for HGG, one of the challenges that need to be overcome is the blood-brain barrier (BBB) (142, 143). BBB is the body’s defense of protecting the brain and the CNS system from toxins, inflammation and infectious sources present in the blood. While it prevents pathogens from getting into the CNS system, it also prevents the passage of therapeutic agents into the brain. Although once considered to be an immuno-privileged site, the CNS is no longer considered to be so with regards to the passage of immune cells (142–144). It is now understood that immune cell trafficking across the BBB is closely regulated (145). The immune system keeps surveillance *via* lymphatics to the deep cervical lymph nodes but immune cells rarely cross the BBB (145). However, when there are excessive inflammatory signals, a plethora of cells including neutrophils, B and T cells and monocytes can enter the CNS to strive to maintain the brain’s homeostasis and avoid damages from the inflammation (145). The macrophages and microglia make up the CNS resident immune system and respond to even minor changes in the CNS homeostasis becoming reactive and causing inflammation (146). There are also immunosuppressive cytokines such as TGFβ or IL-10 that get secreted and encourage further tumor growth making treatment complicated (11).

MRI in adult GBM not only shows the accumulation of normally brain-impermeable material in all GBM, providing evidence of BBB disruption, but also demonstrates that most GBMs have gross tumor burden protected by an intact BBB, suggesting the BBB is still a key barrier to effective treatment (147, 148).

In the case of pediatric tumors, different subgroups affect the BBB differently (149–151). It has been noted that DMGs display little to no contrast enhancement on MRI compared to other pHGGs, suggesting a more intact BBB (149, 150). This is probably due to other pHGGs induce abnormal vessel morphology and BBB disruption while DMGs maintain a vasculature similar to that of normal brainstem (151).

While the BBB poses a challenge, so does the immunosuppressive TME (152, 153). Berghoff and colleagues found that the status of IDH mutation was associated with the immunosuppressive TME of diffuse glioma; IDH mutant diffuse gliomas including GBM had a lower rate of T cell infiltration than the IDH-wildtype cases (154). In addition, the glioma TME is characterized by hypoxic areas and low nutrients which causes autophagy and response to stress that hamper T cell function (155, 156). Indoleamine 2,3 dioxygenase (IDO) and arginase (Arg1) highly expressed by tumor cells and myeloid cells within the TME can cause suppression of T cell functions (157, 158). Tumor cells and macrophages in hypoxic conditions also release immunosuppressive factors such as hypoxia‐inducible factor‐1 alpha (HIF‐1α) and prostaglandin E2 (PGE2) and inhibit T cell proliferation and function (159, 160). Targeting these hypoxia and metabolic pathways may alter the immunosuppressive TME and enhance T or CAR T cell therapy (161).

Tumor-associated macrophages/microglia (TAMs) and MDSCs are important components and main proportion of infiltrating immune cells in both adult and pediatric HGG TME (162, 163). Together with the immune-suppressive cytokines and chemokines, these cells prevent the innate and adaptive immune system from recognizing and eradicating the tumor cells (162, 163). High levels of MDSCs are correlated with poor prognosis in both adult and pediatric patients with HGG (135, 164). Interestingly, a negative correlation was reported between the abundance of TAMs and the overall survival in adult but not pediatric HGG patients (165). Furthermore, TAMs in aHGG and pHGG TME seem to present different phenotypes. Lin et al. recently found that although DIPG tumor cells produce CSF1, which is associated with the M2 phenotype of macrophages, macrophages in DIPG TME do not appear to have the characteristics of M2 macrophages as in aHGG (164, 166). Infiltration of cytotoxic effector immune cells including CD8+ T cells and NK cells has been demonstrated in the TME of aHGG; however, the activity of these infiltrating effector cells is suppressed by mechanisms mediated by TGF-β, LDH5, and galectin-1 among others (163, 167). In contrast, scarce T cell infiltration was demonstrated in pHGG TME (153). The tumors harbor histone mutants K27M and G34R were considerably “cold”, based on the observation of lack of CD8+ T cells in immunohistochemical staining (168). Jha et al. investigated the frequency of T cells in both adult and pediatric DMG TME. It was found that while CD3+ T cell frequency did not exhibit much difference, CD8+ T cells showed a significantly greater frequency in adult compared to pediatric DMG TME (169). The same group also evaluated PD-L1 expression in 126 DMG samples from both adult and pediatric patients and found a significant correlation between PD-L1 expression and number of tumor-infiltrating lymphocytes in both age groups. Patients with low PD-L1 expressing tumors had a longer overall survival compared to those with high PD-L1 expressing tumors in both groups (169). All these findings suggest that pHGG and aHGG TMEs are similar, but each has their own unique features.

A challenge of immunotherapy for HGG is tumor antigen heterogeneity both between patients and within individual tumors. Barish et al. investigated the expression of three clinically relevant tumor antigens, IL13Rα2, HER2, and EGFR, using IHC combined with digital imaging and pixel-level quantitative measurements of DAB (3,3′-Diaminobenzidine) visualized immunoreactivity, on tumor samples from 43 aHGG patients who had not been treated with immunotherapy (170). It was observed that expression of these antigens was highly variable and non-homogeneous across all 43 patient samples. Within individual tumor samples, expression of these antigens was not uniform, but rather mapped into local neighborhoods with high or low antigen expression (170). In pHGG patient-derived orthotopic xenograft samples, the expression of HER2, IL-13Rα2, EphA2, B7-H3, and GD2 was evaluated and demonstrated to be heterogenous by flow cytometry (171). In a small-scale study, GD2 expression was investigated by IHC and showed a varying intensity and staining pattern (cytoplasmic/nuclear, focal/diffuse) in tissue samples from 9 pHGG patients (172). The inter-patient tumor antigen heterogeneity hinders the development of a universal single immunotherapy for HGG while the intra-patient heterogeneity results in the survival of tumor antigen-deficient clones in patients treated with single immunotherapy. Immunotherapies targeting multiple antigens simultaneously are explored to overcome tumor antigen heterogeneity in HGG (173, 174).

With the clinical application of cancer immunotherapy comes the increasing awareness of one inherent limitation of immunotherapy, which is the potentially fatal toxicities including cytokine release syndrome (CRS), immune effector cell-associated neurotoxicity syndrome (ICANS), and on-target off-tumor toxicity, among others. CRS is due to high level immune activation by natural and bispecific antibody or CAR T cell which results in massive release of cytokines including IL-6 and IFN-γ, leading to life-threatening side effects (175, 176). ICANS is a neurologic complication caused by CAR T cells. Patients with severe ICANS demonstrate evidence of endothelial activation and BBB disruption (177, 178). On-target off-tumor toxicity results from antigen-specific attack by the immunotherapy on host tissues when the targeted tumor antigen is also expressed on normal tissues. This toxicity can occur after immune checkpoint blockade treatment or adoptive T cell therapy with genetically engineered T cells (179, 180). These toxicities need to be managed by carefully designing the immunotherapy agents and the clinical trials in both adult and pediatric patients (181, 182).

## 7 Conclusion

pHGG is characterized by a specific set of genetic and epigenetic aberrations different from those found in aHGG and therefore is a distinctly different biological disease compared to aHGG (4)(**Table 1**). Molecular targeted therapies that are based on genetic and epigenetic alterations in aHGG are not as effective against pHGG (32–35). A better understanding of the signaling pathways and molecular modulators of pHGG is essential to allow newer specific targeted molecular and other treatment strategies to emerge for pHGG. However, pHGG is a rare disease which poses a challenge to obtain clinical samples for extensive investigation for disease mechanism. However, efforts are underway through research consortiums such as the Children’s Oncology Group and the Childhood Brain Tumor Tissue Network to obtain samples and extensively profile them in order to accelerate research and discovery in this domain. Immunotherapy is a rapidly advancing and promising therapeutic option for HGG. Although pHGG and aHGG are distinctive in terms of molecular biology background, they do harbor some of the same tumor targets such as GD2 (183) and EGFRvIII (128) and have somewhat similar TME with abundant immunosuppressive MDSCs and TAMs and lack of active immune effector cells (135, 162–164, 169), suggesting that certain immunotherapies for aHGG could be extended to pHGG. However, distinctions between aHGG and pHGG as well as the fundamental differences between adult and children need to be recognized when applying adult immunotherapy regimens or developing specific new immunotherapy in pHGG (2, 184, 185). Significant challenges remain, these include delivery of immunotherapy reagents to penetrate BBB, overcoming tumor heterogeneity, managing immunotherapy associated toxicities and circumventing immunosuppressive TME. Carefully designed combinatorial therapy is likely to be the key to addressing these challenges. With rapid scientific advances being made and a number of ongoing and soon-to-follow clinical trials, more effective therapies are promised to change the future course and improve the outcome of the adults and children with these devastating tumors.

## Author contributions

PA, WL, KP, HH, PR, TC, KC, DL, and MC wrote, reviewed and revised the manuscript. All authors approved the final manuscript for submission.

## Funding

This work was supported in part by grant U54 (CA232561-01/A1) (MC, TC, DL, KC), Pediatric Cancer Research Foundation (MC) and Children’s Cancer Fund (MC).

## Conflict of interest

MC serves as a consultant for Jazz, Omeros, Astra Zeneca, Nektar Therapeutics and Novartis and a member of the Speakers Bureau for Jazz, Servier, Amgen, Sanofi and Sobi. DL reports personal fees and other from Kiadis Pharma, CytoSen Therapeutics, Courier Therapeutics, and Caribou Biosciences outside the submitted work; In addition, DL has a patent broadly related to NK cell therapy of cancer with royalties paid to Kiadis Pharma. TC recently served as a one-time consultant to Blueprint, Incyte, Oncopeptides, serves as a DSMB chair for SpringWorks and is a cofounder of Vironexis Biotherapeutics, Inc.

The remaining authors declare that the research was conducted in the absence of any commercial or financial relationships that could be construed as a potential conflict of interest.

## Publisher’s note

All claims expressed in this article are solely those of the authors and do not necessarily represent those of their affiliated organizations, or those of the publisher, the editors and the reviewers. Any product that may be evaluated in this article, or claim that may be made by its manufacturer, is not guaranteed or endorsed by the publisher.

## Glossary

**Table:**

HGG	High Grade Glioma
GBM	Glioblastoma Multiforme
CNS	Central Nervous System
MRI	Magnetic Resonance Imaging
EGFR	Epidermal Growth Factor Receptor
PTEN	Phosphatase and Tensin homolog Gene
IDH	Isocitrate Dehydrogenase
TCA	Tricarboxylic Acid Cycle
BRAF	Serine/ Threonine-protein kinase B-Raf
PDGFR	Platelet-Derived Growth Factor Receptor
BBB	Blood Brain Barrier
TME	Tumor Microenvironment
CTLs	Cytotoxic T Lymphocytes;
WT1	Wilms Tumor protein 1
CMV	Cytomegalovirus
pp65	Phosphoprotein 65
EphA2	Ephrin type-A receptor 2
IL13Ra2	Interleukin-13 Alpha 2
DCV	Dendritic Cell Vaccines
MAGE-1	Melanoma-Associated Antigen-1
AIM-2	Antigen isolated from Immunoselected Melanoma-2
TRP-2	Tyrosine-Related Protein-2
gp100	Glycoprotein 100
HSV-1	Herpes Simplex type 1 Virus
DIPG	Diffuse Intrinsic Pontine Glioma
ICB	Immune Checkpoint Blockade
CTLA-4	Cytotoxic T Lymphocyte-associated Antigen 4
PD-1	Programmed cell Death protein 1
PD-L1	Programmed cell Death protein ligand 1
MSI	Microsatellite Instability
ADC	Antibody and antibody Drug Conjugates
NK	Natural Killer cells
mAbs	Monoclonal Antibodies
ADCC	Antibody Dependent Cell mediated Cytotoxicity
CAR	Chimeric Antigen Receptor;
DMG	Diffuse Midline Gliomas
C7R	Interleukin-7 Receptor;
KIRs	Killer Inhibitory Receptors
CRS	Cytokine Release Syndrome
ICANS	Immune effector Cell-Associated Neurotoxicity Syndrome
MDSCs	Myeloid-Derived Suppressor Cells
iNOS	Inducible Nitric Oxide Synthase
TK	Thymidine Kinase
Flt3L	Fms-like Tyrosine Kinase Ligand;
IDO	Indoleamine 2 3 Dioxygenase
Arg1	Arginase
HIF-1a	Hypoxia-Inducible Factor-1 Alpha
PGE2	Prostaglandin E2;
TAMs	Tumor-Associated Macrophages/Microglia
DAB	3,3’-Diaminobenzidine.
